# User Perspectives of Diet-Tracking Apps: Reviews Content Analysis and Topic Modeling

**DOI:** 10.2196/25160

**Published:** 2021-04-22

**Authors:** Mila Zečević, Dejan Mijatović, Mateja Kos Koklič, Vesna Žabkar, Petar Gidaković

**Affiliations:** 1 School of Economics and Business University of Ljubljana Ljubljana Slovenia; 2 Zuehlke Engineering Schlieren Switzerland

**Keywords:** diet-tracking apps, mobile apps, user reviews, topic modeling, n-grams, mHealth, nutrition, diet, well-being

## Abstract

**Background:**

The availability and use of mobile apps in health and nutrition management are increasing. Ease of access and user friendliness make diet-tracking apps an important ally in their users’ efforts to lose and manage weight. To foster motivation for long-term use and to achieve goals, it is necessary to better understand users’ opinions and needs for dietary self-monitoring.

**Objective:**

The aim of this study was to identify the key topics and issues that users highlight in their reviews of diet-tracking apps on Google Play Store. Identifying the topics that users frequently mention in their reviews of these apps, along with the user ratings for each of these apps, allowed us to identify areas where further improvement of the apps could facilitate app use, and support users’ weight loss and intake management efforts.

**Methods:**

We collected 72,084 user reviews from Google Play Store for 15 diet-tracking apps that allow users to track and count calories. After a series of text processing operations, two text-mining techniques (topic modeling and topical n-grams) were applied to the corpus of user reviews of diet-tracking apps.

**Results:**

Using the topic modeling technique, 11 separate topics were extracted from the pool of user reviews. Most of the users providing feedback were generally satisfied with the apps they use (average rating of 4.4 out of 5 for the 15 apps). Most topics referred to the positive evaluation of the apps and their functions. Negatively rated topics mostly referred to app charges and technical difficulties encountered. We identified the positive and negative topic trigrams (3-word combinations) among the most frequently mentioned topics. Usability and functionality (tracking options) of apps were rated positively on average. Negative ratings were associated with trigrams related to adding new foods, technical issues, and app charges.

**Conclusions:**

Motivating users to use an app over time could help them better achieve their nutrition goals. Although user reviews generally showed positive opinions and ratings of the apps, developers should pay more attention to users’ technical problems and inform users about expected payments, along with their refund and cancellation policies, to increase user loyalty.

## Introduction

Obesity and overweight are the result of a plethora of environmental factors that are known to influence individuals’ food intake and physical activity [1,2]. As obesity is a global public health challenge that leads to numerous health, social, and economic difficulties [3], it is important to help individuals make better and healthier dietary choices. Changing lifestyles (eg, the modern sedentary lifestyle) along with other features of an obesogenic environment (eg, physical design challenges; political, social, and cultural factors; the (in)ability to promote an active lifestyle; better food choices) have created a complex environment for consumers to manage their weight and diet [4,5]. Despite this dysfunctional environment, people are aware of the negative impact that inappropriate diets can have on their health and well-being, and are generally interested in making healthier choices and educating themselves about the foods they consume and their nutritional value [6].

Technological advancements, including those related to mobile devices, are enabling developments of an increasing number of tools to help individuals take control of their health and nutrition. Mobile apps have become an important source of information for their users. A study conducted in the United States showed that more than half of the population using a mobile phone has downloaded at least one health-related app. Most of these were fitness and nutrition apps, which also have the highest reported usage rates [7,8].

Particularly in the area of nutrition, people use apps for a variety of purposes, including to learn about products, read or contribute recipes, interact in app community forums, evaluate their food choices, check product labels, and obtain an overview of the healthiness of products [9], as well as to track their food intake and diets [8]. Given the importance of self-monitoring and diet tracking in motivating behavior change and persistence in healthier eating [8,10], we have been focusing on the nutrition apps that provide a calorie-tracking option (ie, diet-tracking apps). These apps promote diet tracking and maintenance of healthier habits and do not necessarily include weight loss as a goal, which has often been the focus of previous studies (eg, [7]).

The low inclusion of behavior change strategies in diet tracking apps may hinder their ability to help users achieve their long-term diet and nutrition goals [11,12]. However, diet-tracking apps that successfully employ behavior change strategies can have a positive effect on their users’ motivation, habits, and diet and nutrition outcomes [13-16]. These apps have also proven to be helpful in behavioral control and weight management [15]. Self-monitoring is considered the cornerstone of successful weight management [17], and can be particularly helpful when combined with tailored goals [16]. Goal setting, which diet-tracking apps enable, is one of the relevant factors influencing behavior change, along with motivation and self-efficacy [15]. Together with apps that offer personalized meal planning programs [18], there is evidence that diet-tracking apps can efficiently support behavior change and weight management [18,19].

Nevertheless, the usability of diet-tracking apps in the weight management process has not always been positively assessed. Intervention studies focusing on diet-tracking apps show that users sometimes dislike the apps due to their complexity, lack of personalization and long-term support for users, and the focus on calorie counting, which can easily become an obsession [20]. Maintaining high levels of motivation throughout the weight management journey is challenging for most people, and diet-tracking apps sometimes fail to provide proper motivational support for sustainable weight loss and weight management [20,21]. In addition, the long-term effects of diet-tracking apps on food intake and user behavior remain unexplored [16]. These issues indicate that additional research on diet-tracking apps and their features is needed [7].

As consumers increasingly rely on apps to support their daily activities, they also generate invaluable feedback for both developers and potential users through app reviews and ratings. These reviews typically contain information that is valuable for app evaluation, including user opinions about the app, information about their experiences with the app, and bug complaints or feature suggestions [22]. A previous study showed that almost a quarter (23.3%) of app reviews contain an app feature request or app assessment [23].

App developers and companies have recognized the value of user reviews and frequently examine these reviews to improve the user experience. Most mobile health apps are free to use, with the option to upgrade profiles to paid premium options (for more features or personalized advice). The initial free download and use make these apps easily disposable and users’ decision to switch between them is widespread [24]. This drives app developers’ interest in listening to users’ comments, complaints, ideas, and suggestions [25]. In reviews, users are text producers for other potential consumers, businesses, and society at large. This text can then be used to predict and understand user preferences and behaviors [26].

To increase usage and enable better health and nutrition outcomes, it is necessary to better understand user needs. It is evident that users are not simply looking for pure information when using mobile health apps. The way the information is presented; the usability of the app; the degree to which it engages and connects users; and its effectiveness, timeliness, design, and functionality are also important considerations [27]. A deeper analysis of user reviews of apps can lead to better knowledge of desired features and user preferences, and ideally increase the usability, appeal, and effectiveness of the app in achieving users’ health and nutrition goals [7]. These aspects require more academic research into user reviews [22], especially as functionality and appearance often have greater contributions to the popularity of dieting apps than the quality of the information provided by the app [28].

Owing to their presence and relevance in the dieting field, diet-tracking apps have attracted the interest of many researchers who have used app evaluation strategies in an attempt to better understand and evaluate app features [29-32]. The influence of diet-tracking apps on users’ food choices and their opinions about these apps have been tested using experimental and survey data collection methods [10,15,18,33]. Previous studies also assessed the consistency of information provided by different apps, and recommended further collaboration and harmonization of information [34,35].

To improve the understanding of user opinions on diet-tracking apps, this study was performed based on the collection and analysis of the textual reviews and numerical ratings that users leave for these apps. We focused on identifying the main positive and negative aspects that users express about diet-tracking apps and openly share in app reviews. By identifying the features or functions that attract consumers’ attention, this study suggests areas for app development and improvement that have potential to increase users’ positive evaluation and motivation, leading to nutrition/health improvement.

## Methods

### Data Collection

To evaluate users’ views on diet-tracking apps, we used a different methodology from the experimental and survey data collection methods previously reported [10,15,18,33]. We performed a thorough content analysis of the app user reviews available on Google Play Store. The text mining method was used to detect the word and topic combinations that are frequently mentioned in user reviews. These word combinations (n-grams) were evaluated and the most frequent combinations were selected for further analysis. The identified topics were named and are briefly discussed.

The search for diet-tracking apps in Google Play Store included the following keywords: “nutrition apps,” “diet apps,” “calorie counter apps,” “food scanner,” “calorie app,” “calorie tracker,” and “calorie scanner.” We identified 131 unique apps that offer calorie information (eg, calorie tables, calorie tracking, diet diaries). These apps were further reviewed to determine if they offered a calorie counting option for food items and an intake diary for users, and if their operating language was English. Final app selection was based on the number of downloads and reviews (impact evaluation; apps with over 1 million downloads and at least 10,000 user ratings were selected), which is a standard procedure in the app assessment process (eg, [7,30]). A total of 15 apps were identified that met all of the above conditions (Table 1), and their reviews were scraped from Google Play Store using a custom-written Python script. In total, 81,660 of the latest user reviews for these apps were collected at this stage.

**Table 1:** Overview of apps included in the study.

App	Mean app rating	Number of downloads	Number of ratings
Health Pal-Fitness, Weight Loss Coach, Pedometer	4.1	>1 million	>20,000
iEatBetter: Food Diary	4.2	>1 million	>20,000
Health & Fitness Tracker with Calorie Counter	4.1	>1 million	>20,000
Calorie Counter by Fat Secret	4.7	>10 million	>300,000
Calorie Counter by Lose It!	4.6	>10 million	>100,000
Fooducate-Eat better. Lose weight. Get healthy.	4.4	>1 million	>15,000
Calorie Counter-MyNetDiary, Food Diary Tracker	4.6	>1 million	>40,000
Healthify me-Calorie Counter, Weight Loss Coach	4.5	>10 million	>100,000
MyPlate Calorie Tracker	4.6	>1 million	>30,000
Calorie Counter-My Fitness Pal	4.4	>50 million	>2 million
Lifesum-Diet Plan, Macro Calculator & Food Diary	4.4	>10 million	>200,000
Noom: Health & Weight	4.4	>10 million	>200,000
YAZIO Calorie Counter, Nutrition Diary & Diet Plan	4.6	>10 million	>300,000
Calorie, Carb & Fat Counter	4.5	>1 million	>50,000
Calorie Counter Calories!	4.5	>1 million	>10,000

### Data Analysis

#### Text Mining Approaches

With the increase in publicly available user-generated content due to the proliferation of internet-assisted communication, researchers have developed several automated approaches to identify, summarize, and classify the available information [26,36]. The development of new tools allows researchers to obtain more information about users’ opinions and sentiments in their writing. There is a trend to shift the focus of opinion mining from studying long texts to shorter user posts on various social media platforms and websites [22]. The rapid development and increase in the efficiency and capabilities of text mining and natural language processing (NLP) algorithms is evident, and they are becoming an important part of social science research in the study of user-generated content [37,38]. Speed, reproducibility, and reliability are considered some of the most important advantages of text mining when it comes to classifying and categorizing text [39].

In this study, we applied two text mining methods to our dataset: topical n-grams identification and topic modeling. Both methods work by identifying and grouping words that occur simultaneously in the text (user reviews in our case). Data analysis required preprocessing of the raw data, which was performed through several procedures commonly used in data preparation and preprocessing for text mining analysis [40].

#### Data Preprocessing

Data preprocessing is a data mining technique that transforms raw data into an understandable format. Real-world data are often incomplete, inconsistent, contain a substantial amount of redundant information, and are likely to include many errors [41]. This is a critical and time-consuming process as the output depends on the quality of the data.

Since both the topical n-grams identification and topic modeling approaches have the same preprocessing steps, the same preprocessed dataset was used in both methods. For these tasks, we used the Python programming language in combination with its data science–specific tools (ie, libraries) that made this process possible given the large amount of data. The Python libraries numpy and pandas, which are well known in the data science community, were used extensively throughout the process, along with several other libraries, each specialized for a particular task. The following data preprocessing steps were applied.

First, we removed all non-English reviews. We were only interested in English reviews at this point since our dataset contains reviews in different languages such as Portuguese, Spanish, and German. These reviews make up about 12% of our dataset (9576 reviews), and it was safer to remove them than to translate them into English. A total of 72,084 user reviews in English were identified in this step using the Python library langdetect. These reviews were used for all analyses performed in this study.

We then converted the text to lowercase, performed an extensive spell check of every review, and made necessary corrections using the Speller Python library. Words such as “I,” “are,” “and,” and “the” were considered “stop words” and removed, as such common words tend to dominate the results. We further removed any special characters and numbers from the reviews.

Second, lemmatization was performed, which is a process of grouping the inflected forms of words so that they can be analyzed as a single item. These forms are identified by the lemma of the word (ie, both “tracking” and “tracks” share the same lemma and become “track”). The lemmatization algorithm considers the morphological analysis of the words during data preparation [42]. Finally, we applied part-of-speech tagging, which determines the category of the word (eg, noun, adjective, adverb, verb) based on both its definition and context [43].

After data preprocessing, a new dataset was obtained with cleaned data that could be used for both topic modeling and n-grams identification. This new dataset comprised a collection of arrays of words. For example, the item in the previous dataset *‘*’I love this app and it’s easy to use’’ is converted into the word array “love app easy use,” which was used as such for further analysis.

#### Topical N-Grams Identification

The use of topical n-grams is common in text and topic mining, as is the NLP approach when tracking word or phrase frequencies [44,45]. For the purpose of this study, we implemented the analysis of the most frequent trigrams (ie, groups of three words used together) occurring in user reviews (textual user evaluation of the app). For example, applying this to our previously mentioned word array from the cleaned dataset *“*love app easy use,” we could extract exactly two trigrams: (love, app, easy) and (app, easy, use).

These two trigrams would then be added to the trigrams extracted from other reviews, resulting in a total of 744,808 trigrams from 72,084 reviews.

Evaluation of the word combination was then used in conjunction with the users’ numerical app assessment (ie, rating), which is often used as a proxy for sentiment (eg, [46,47]). User ratings for apps in Google Play Store are scored on a scale from 1 to 5, where 1 is the worst and 5 is the best rating an app can receive. In our study, scores below 3 were associated with a negative app assessment, whereas scores of 4 and 5 were considered positive. This approach is common in studies in this field (eg, [46]). The ratings were averaged by grouping ratings from reviews containing given trigrams.

#### Topic Modeling

Topic modeling is another text-mining and NLP method that is commonly used to discover latent topics in a corpus of text. Topic modeling has been shown to be useful for clustering documents or text, and is considered a probabilistic statistical technique for semantic structures [48]. In this study, we used the Python Gensim library, which is commonly used in NLP, for topic modeling analysis. This library helped us to build a mathematical model that could classify each review by topic. The list of possible topics was determined during model training, and we predetermined the number of possible topics. To find the most appropriate number of topics, we used the coherence score. Topic coherence measures the degree of semantic similarity between the highly scored words in the topic, which can help to distinguish between topics that are semantically interpretable and topics that are artifacts of statistical inference [49]. This value is given after each model training process and helped us determine the performance of our trained model.

Finding the best number of topics that would give optimal results required several trials, starting with a randomly selected number of topics until we narrowed down to the model with the best score. We would simply select this model and apply it to our dataset. For example, if our model found 11 topics in the dataset, for each review in our dataset, the model would provide us with the probabilities of how likely the review is to belong to each of the 11 topics.

## Results

### Topic Modeling

#### Topic Selection Process

**Figure 1:**
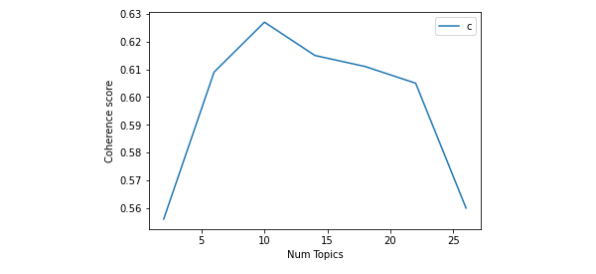
Topics coherence score (range between 2 and 30 topics). Num: number.

**Figure 2:**
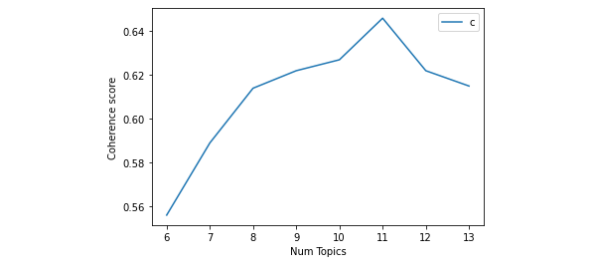
Topics coherence score (range between 6 and 13 topics). Num: number.

Since topic modeling is an unsupervised method, it was not constrained by certain predefined standards (ie, number of topics). Instead, for the first run, we programmed the script to start with only 2 topics, repeat and increase by 4 (since this is a computationally intensive and demanding process, we had to minimize the number of runs) until reaching 30 (ie, finding optimal number of topics anywhere between 2 and 30 topics). This high number was randomly chosen to find the optimal range for our topic number. This analysis revealed that the number of topics with the best coherence score was between 6 and 13 (Figure 1). For the second iteration, we again reprogrammed the script to repeatedly run in this range, this time increasing by 1 to more accurately determine the best score. The best coherence (0.646) was achieved with 11 topics (Figure 2, Table 2).

**Table 2:** Coherence scores for 6 to 13 topics.

Number of topics	Coherence score
6	0.59
7	0.618
8	0.617
9	0.614
10	0.614
11	0.646
12	0.618
13	0.622

#### Identified Topics

Most of the identified topics included the use of positive words when describing apps in the reviews. In their feedback, users often use words such as “love,” “nice,” “easy,” “good,” and “amaze” to describe the apps. Positively rated topics were more common than negatively rated topics. Users who leave feedback for diet-tracking apps positively rate the possibility to track their food intake; use food scanners and create/access food databases in the apps; and consider the apps to be user-friendly, convenient, and easy to use overall. Weight loss was another important topic, appearing in 10% of user reviews (Table 3).

By pairing the topics with the average ratings of user reviews from the topic, we found that difficulties with cancellations, payment plans, and charges seem to bother users the most (topic average rating 2.32). One of the users described her experience as follows:

Personally I felt company was highly interested in my weight loss journey before I activated plan then no one cares about me, or I noticed they've charged me for another 3 months subscription which I did not authorize. I've made 3 attempts via email to make contact with [app] to end my membership and request a refund and I received an email back stating they'll be in contact in the next 48hrs but I have never heard back from them.

In addition, technical difficulties appear to create issues in using the app (average topic rating 2.84):

Good app when it works. Otherwise, there's too many bugs. It lags too often and takes a long time to load…

Stopped working. When I try to add food or search it's a blank screen. Please fix!

**Table 3:** Modeling results for 11 selected topics.

Topic	Words	Mean rating	Proportion of reviews (%)
Health and fitness tracking	App, great, work, good, health, step, fitness, tracker, sync, google	4.413731	10.00
Macros tracking	App, great, love, track, awesome, carbs, fat, diet, macro, feature	4.590072	8.13
App praising	App, good, food, thing, lot, find, put, log, info, pretty	4.342717	7.34
App support	Give, room, program, coach, support, day, information, follow, people, plan	4.264332	7.21
App charges	Free, pay, version, plan, premium, cancel, money, charge, month, trial	2.329310	8.96
Weight loss	Weight, lose, week, goal, loss, start, pound, year, month, set	4.697312	10.94
Intake tracking	Calorie, track, exercise, intake, count, daily, day, water, great, burn	4.578164	10.06
Food adding and database	Food, add, meal, option, item, database, recipe, enter, list, search	3.872253	8.77
App “loving”	Easy, love, food, helpful, scan, user, simple, find, scanner, feature	4.709999	12.67
Diet change	Eat, make, change, diet, healthy, recommend, choice, learn, life, habit	4.780649	8.37
Technical issues	Time, work, log, update, day, star, back, phone, issue, problem	2.848619	7.53

### Topical Trigrams

#### Overall Ratings

Similar to the topic modeling results, our overall trigram analysis suggested that, on average, users rate the diet-tracking apps positively in their reviews (Table 4). Trigrams indicating the apps’ ease of use and helpfulness (21/50 top trigrams by frequency of mention), the expression of liking/loving the app (13/50 top trigrams by frequency of mention), and the apps’ tracking option (19/50 top trigrams by frequency of mention) dominated the top 50 trigrams identified from the pool of user reviews. These topics were also frequently associated with high average app ratings; 49 of the top 50 trigrams by frequency attained ratings over 4.

**Table 4:** Top 50 most frequently mentioned trigrams.

Trigram	Count	Mean rating	Category
(app, easy, use)	948	4.772152	Easy Use/Help
(help, keep, track)	895	4.773184	Easy Use/Help; Tracking
(keep, track, calorie)	591	4.626058	Tracking
(help, lose, weight)	457	4.628009	Easy Use/Help; Weight Loss
(help, stay, track)	345	4.837681	Easy Use/Help; Tracking
(app, keep, track)	341	4.750733	Tracking
(really, like, app)	309	4.281553	App Liking
(app, really, help)	307	4.856678	Easy Use/Help
(track, calorie, intake)	303	4.669967	Tracking
(keep, track, eat)	296	4.733108	Tracking
(easy, use, love)	290	4.858621	Easy Use/Help; App Liking
(track, food, intake)	283	4.614841	Tracking
(keep, track, food)	263	4.653992	Tracking
(app, track, calorie)	258	4.701550	Tracking
(love, app, help)	246	4.857724	Easy Use/Help; App Liking
(love, app, easy)	244	4.831967	Easy Use/Help; App Liking
(great, app, track)	241	4.726141	App Liking; Tracking
(great, app, easy)	239	4.861925	App Liking; Easy Use/Help
(bar, code, scanner)	236	4.199153	N/A^a^
(app, help, keep)	235	4.804255	Easy Use/Help; Tracking
(great, app, help)	226	4.774336	App Liking; Easy Use/Help
(easy, use, great)	224	4.843750	Easy Use/Help
(way, keep, track)	222	4.729730	Tracking
(easy, use, helpful)	216	4.847222	Easy Use/Help
(really, help, keep)	204	4.803922	Easy Use/Help; Tracking
(make, good, choice)	204	4.779412	N/A
(weight, loss, journey)	201	4.731343	Weight Loss
(use, app, year)	201	4.129353	N/A
(easy, keep, track)	201	4.850746	Easy Use/Help; Tracking
(app, help, lose)	200	4.755000	Weight Loss; Easy Use/Help
(easy, use, help)	191	4.858639	Easy Use/Help
(really, easy, use)	188	4.781915	Easy Use/Help
(weight, loss, program)	186	4.688172	Weight Loss
(super, easy, use)	180	4.911111	Easy Use/Help
(easy, use, keep)	173	4.809249	Easy Use/Help; Tracking
(great, app, keep)	171	4.719298	App Liking; Tracking
(easy, use, app)	171	4.666667	Easy Use/Help
(keep, track, everything)	169	4.869822	Tracking
(really, good, app)	169	4.426036	App Liking
(use, free, version)	165	4.230303	NA
(good, app, track)	163	4.595092	App Liking; Tracking
(weight, loss, goal)	161	4.714286	Weight Loss
(start, use, app)	160	4.387500	N/A
(scan, bar, code)	158	4.227848	N/A
(try, lose, weight)	157	4.414013	Weight Loss
(use, keep, track)	154	4.779221	Tracking
(absolutely, love, app)	153	4.790850	App Liking
(love, app, use)	144	4.472222	App Liking
(would, give, star)	144	3.638889	N/A
(best, app, ever)	142	4.929577	App Liking

^a^N/A: not applicable; no relevant category.

The number of mentions of positive and negative trigrams in user reviews also showed a trend of positive evaluation dominance among users leaving reviews. The top 50 most frequent positive trigrams appeared 12,723 times, while the top 50 most frequent negative trigrams were mentioned 1270 times in our dataset of 72,084 user reviews.

#### Positively Evaluated App Characteristics

With respect to positively valenced user ratings, we identified a significant presence of reviews praising the apps in general. Most of the top 50 positively rated user reviews refer to the apps’ ease of use and helpfulness (21/50 top positive trigrams by frequency of mention), intake and calorie tracking (20/50 top positive trigrams by frequency of mention), and weight loss (6/50 top positive trigrams by frequency of mention) (Table 5). Users generally characterized the apps as “very easy to use and intuitive” and “easy to use and pretty straightforward.” The ability to track their intake is described as “keeps me accountable” and “very informative about the diets you choose.” Some users also viewed the apps as contributors to their better and healthier food choices:

Helps get a better understanding of the different foods calorie loads so I can make better choices.

Easy to check total carb, fat and protein content and individual food values so I can make better choices next day.

Similar comments were found for weight loss:

Teaches you how and why you need to change your eating habits;

Mind changing weight loss program;

This is about sustainable weight loss...

**Table 5:** Top 50 “positive” trigrams (most frequently mentioned trigrams with ratings over 4).

Trigrams	Count	Mean rating	Category
(app, easy, use)	948	4.772152	Easy Use/Help
(help, keep, track)	895	4.773184	Easy Use/Help; Tracking
(keep, track, calorie)	591	4.626058	Tracking
(help, lose, weight)	457	4.628009	Easy Use/Help; Weight Loss
(help, stay, track)	345	4.837681	Tracking; Easy Use/Help
(app, keep, track)	341	4.750733	Tracking
(really, like, app)	309	4.281553	App Liking
(app, really, help)	307	4.856678	Easy Use/Help
(track, calorie, intake)	303	4.669967	Tracking
(keep, track, eat)	296	4.733108	Tracking
(easy, use, love)	290	4.858621	Easy Use/Help; App Liking
(track, food, intake)	283	4.614841	Tracking
(keep, track, food)	263	4.653992	Tracking
(app, track, calorie)	258	4.701550	Tracking
(love, app, help)	246	4.857724	Easy Use/Help; App Liking
(love, app, easy)	244	4.831967	Easy Use/Help; App Liking
(great, app, track)	241	4.726141	App Liking; Tracking
(great, app, easy)	239	4.861925	App Liking; Easy Use/Help
(bar, code, scanner)	236	4.199153	N/A^a^
(app, help, keep)	235	4.804255	Easy Use/Help; Tracking
(great, app, help)	226	4.774336	Easy Use/Help; App Liking
(easy, use, great)	224	4.843750	Easy Use/Help
(way, keep, track)	222	4.729730	Tracking
(easy, use, helpful)	216	4.847222	Easy Use/Help
(really, help, keep)	204	4.803922	Easy Use/Help; Tracking
(make, good, choice)	204	4.779412	N/A
(weight, loss, journey)	201	4.731343	Weight Loss
(use, app, year)	201	4.129353	N/A
(easy, keep, track)	201	4.850746	Easy Use/Help; Tracking
(app, help, lose)	200	4.755000	Weight Loss; Easy Use/Help
(easy, use, help)	191	4.858639	Easy Use/Help
(really, easy, use)	188	4.781915	Easy Use/Help
(weight, loss, program)	186	4.688172	Weight Loss
(super, easy, use)	180	4.911111	Easy Use/Help
(easy, use, keep)	173	4.809249	Easy Use/Help; Tracking
(great, app, keep)	171	4.719298	App Liking; Tracking
(easy, use, app)	171	4.666667	Easy Use/Help
(keep, track, everything)	169	4.869822	Tracking
(really, good, app)	169	4.426036	App Liking
(use, free, version)	165	4.230303	N/A
(good, app, track)	163	4.595092	App Liking; Tracking
(weight, loss, goal)	161	4.714286	Weight Loss
(start, use, app)	160	4.387500	N/A
(scan, bar, code)	158	4.227848	N/A
(try, lose, weight)	157	4.414013	Weight Loss
(use, keep, track)	154	4.779221	Tracking
(absolutely, love, app)	153	4.790850	App Liking
(love, app, use)	144	4.472222	App Liking
(best, app, ever)	142	4.929577	App Liking
(app, track, food)	142	4.549296	Tracking

^a^N/A: not applicable; no relevant category.

#### Negatively Evaluated App Characteristics

Based on our results, an aggressive approach to advertising premium app options, and unclear policies of subscription charges and cancellations seem to be particularly problematic (15/50 top negative trigrams by frequency of mentions). The reviews also indicate that constant display of ads and reminders about premium app options often lead users to delete the app, as illustrated by the following excerpts from the reviews:

Every time I open the app it pushes its premium service at me. I get that they are here to make money, but seriously, just throw a nonintrusive ad window in somewhere and don't pester me.

Go for alternatives until these guys stop giving you ads for paid plans.

NO MONEY BACK NO MATTER WHAT. This app didn’t work for me since I'm not overweight. I tried it to give it a chance thinking it was what I was looking for, but it wasn’t. and then found out that NO MATTER what, you can’t get your money back once they have charged the subscription even if it's on THE SAME DAY.

Given the size of the apps in question and their numbers of users, it is easy to see how the ability to upload content could be difficult to manage from a technical perspective. Nevertheless, 14 of the 50 most frequently mentioned negative trigrams refer to technical issues experienced by users (Table 6), which include app crashing, inability to use or open the app, and similar:

It freezes every time I try to add food or exercise.

Not sure what's going on however ever since paid ads keep popping up the app has just gone down hill. Today in particular has been awful. Screen goes black, freezes. Constant crashing. I've used this app for 3 years now and am seriously looking at using another app.

I really wanted to give this app 5 stars, especially since it's helped me to lose more than 20 pounds in the last 6 weeks. Unfortunately, the app itself is so laggy & buggy that I can't give it more than 1-star. Every time I shift between apps, [app] needs 15-60 seconds to start up. The whole app crashes on me at least 10 times a day.

Some content-related complaints could also be found in reviews, such as “need to be able to add new food with more than the 100 grams” or “needs an option to let users easily add new foods and correct scanned foods that have incorrect nutritional data.”

Adding new foods creates additional issues for users (5/50 top frequent negative trigrams). Namely, users’ complaints in this area usually refer to the inability to add a product to the database due to technical challenges:

There's an option to add a new food item, but no way to save it.

Would have been a perfect app but becomes utterly useless when trying to input my own foods. Everyone I enter the nutritional info it changes everything I put in to insane numbers like 2800 calories for cottage cheese.

The reason I gave this a 4 is because the app doesn't always keep my information I add about new foods. It constantly says it's downloading the database for days on end.

**Table 6:** Top 50 negative trigrams (most frequently mentioned trigrams with ratings lower than 3).

Trigrams	Count	Mean rating	Category
(every, time, try)	75	2.080000	Technical issues
(use, love, app)	71	2.478873	N/A^a^
(get, money, back)	50	1.240000	Charges/Ads
(day, free, trial)	43	1.674419	Charges/Ads
(can, not, get)	40	1.650000	NA
(try, add, food)	39	2.435897	Adding Food
(since, last, update)	35	2.685714	Technical issues
(sign, free, trial)	33	1.303030	Charges/Ads
(every, time, open)	33	2.484848	Technical issues
(time, open, app)	30	2.366667	Technical issues
(can, not, use)	30	1.633333	N/A
(app, keep, crash)	28	2.107143	Technical issues
(even, use, app)	26	1.653846	N/A
(add, food, meal)	26	2.884615	Adding Food
(bad, app, ever)	26	1.000000	N/A
(get, new, phone)	26	2.692308	N/A
(heart, rate, monitor)	26	2.884615	N/A
(pay, monthly, fee)	25	2.560000	Charges/Ads
(want, money, back)	25	1.160000	Charges/Ads
(every, time, go)	25	2.880000	Technical issues
(try, cancel, subscription)	24	1.166667	Charges/Ads
(app, stop, work)	24	1.916667	Technical issues
(waste, time, money)	24	1.166667	N/A
(can, not, add)	24	2.250000	Adding Food
(charge, credit, card)	24	1.041667	Charges/Ads
(never, use, app)	21	1.952381	Adding Food
(app, can, not)	21	2.142857	Technical issues
(every, single, time)	20	2.400000	Technical issues
(wish, could, give)	20	2.250000	N/A
(get, error, message)	19	1.736842	Technical issues
(something, go, wrong)	18	1.388889	Technical issues
(change, serve, size)	18	2.666667	N/A
(try, use, app)	18	1.888889	N/A
(would, great, app)	18	2.944444	N/A
(free, trial, end)	17	1.470588	Charges/Ads
(use, different, app)	17	2.941176	N/A
(use, app, without)	17	2.705882	N/A
(able, use, app)	17	2.470588	N/A
(try, get, refund)	17	1.470588	Charges/Ads
(give, money, back)	17	1.470588	Charges/Ads
(can, not, log)	17	2.058824	Technical issues
(create, new, account)	17	1.294118	Technical issues
(can, not, enter)	17	2.470588	Technical issues
(cancel, free, trial)	17	1.235294	Charges/Ads
(want, cancel, subscription)	16	1.437500	Charges/Ads
(message, goal, specialist)	16	1.875000	N/A
(create, new, food)	16	2.937500	Adding Food
(two, week, trial)	16	2.875000	Charges/Ads
(buy, pro, version)	16	2.812500	Charges/Ads
(ad, pop, every)	15	1.733333	Charges/Ads

^a^N/A: not applicable; no relevant category.

## Discussion

### Principal Findings

Despite extensive literature on nutrition apps and individual usage patterns in health and nutrition research, the investigation of these apps from the perspective of user-generated content (ie, publicly available user reviews) is in its infancy. Previous research has mainly focused on app development issues and feature evaluation to make apps more accessible and user-friendly (eg, [7,31]). Numerous studies have examined consumer opinions of existing apps through surveys, interviews, and qualitative content analysis. This existing research captured an overall positive assessment/evaluation of the available apps (ie, app liking), as well as users’ perceptions of these apps as helpful and easy to use (eg, [8,15,50]). These findings were supported by our results, as was the importance of the diet-tracking feature to users [8] and the deterrent effect of ads [8,15]. In contrast to other studies that have incorporated user reviews to assess user perspectives on diet and nutrition apps using simple research methods to manually evaluate a smaller set of user reviews (eg, [8,50]), this study extends this knowledge, and includes over 70,000 user reviews that were evaluated and classified using text mining and NLP techniques.

In our study, we focused on the user perspective, and aimed to evaluate the diet-tracking apps and their features that are most frequently commented on by users in app reviews. Although users rated the apps they use very highly on average (the overall rating for all apps was 4.4 out of 5, with individual app ratings ranging from 4.1 to 4.7), some features could still be improved to enhance the user experience.

The predominant positivity in comments and reviews left by users online has already been noted in a recent study including 25 online platforms [51]. We can also support these findings for diet-tracking app reviews, as our results suggest that the users leaving reviews for diet-tracking apps generally tend to give positive ratings. The extracted topics show the prevalence of positive words used in reviews to describe apps, as well as the average ratings of user reviews. To provide diet-tracking app users with an even better experience, we recommend paying more attention to the food databases and the features for adding new foods to the app’s database. Users are generally satisfied with the apps’ functionality, while a richer database would make it easier for them to make choices, and they would likely feel more motivated than if they had to search for substitutes or go through long procedures to add a product. This is especially valid for apps that do not offer the option of adding products by taking a picture. As this is also one of the features associated with several complaints about technical issues (ie, difficulty in adding new, personalized food items or food scanning), working on advanced technological solutions, enriching the database, or working on a stronger network of products within food categories could reduce the negative evaluation of user experience.

Hidden costs and inadequate communication about the cost of using the app are some of the main reasons reported for users to stop using an app [8]. We also identified these issues as a source of numerous complaints from users leaving app reviews, along with the users’ inability to get their money refunded. Therefore, to retain users and keep them satisfied and loyal, an app should provide adequate, clear, and respectful communication regarding the costs that may be incurred through app use (eg, paid premium versions, in-app purchases, refund policies).

### Limitations

Although this study provides valuable insight into user opinions, it is not without limitations. Owing to feasibility constraints, we focused on available reviews and introduced a set of constraints that allowed us to structure and summarize the otherwise diverse user-generated content in the form of app reviews. Future research could apply other text-mining approaches for data collection, cleaning, and analysis. In performing similar studies, it may be beneficial to differentiate users and their motivations for using the diet-tracking app. This can be done (to a certain extent) by a deeper investigation of the review content and its sentiment.

The use of additional methods (eg, surveys, focus groups, or interviews) would be necessary to include and understand the opinions of users who do not leave feedback in the form of a review and to generalize the findings to the entire population of diet-tracking app users.

In addition, users from different cultures may have different app needs (eg, product availability, serving size differences, religious and other food restrictions). To ensure the generalizability and applicability of such findings to a specific market, the results should also include analysis of additional apps and reviews (both global and local apps) in the local language.

This study focused on apps that offer their users the ability to count their calories and track their diets (ie, diet-tracking apps). Although these features are present in apps that are widely applicable (ie, nutrition apps), the results obtained in this study cannot be generalized to the entire segment of nutrition apps. The inclusion of additional selection criteria and apps would be necessary to claim broader applicability of the results. Similarly, reviewers (consumers who write reviews) have been shown to differ from other customers in terms of income, education, and purchasing behavior [52]. Since only a small proportion of users provide openly worded text, an analyst should be aware of nonresponse bias and how a reviewer’s sentiments can be directly correlated with the sentiments of previous reviews, which affects the corpus in which posts are public [53]. Hence, our results cannot be generalized to the entire population of app users.

In addition, only apps that had the highest download numbers in the market were selected for this study. This selection was made due to their greater impact and ability to influence more individuals. Moreover, these apps make frequent updates to provide better service to their users, support the growing network of users, and avoid technical issues that are usually the subject of user complaints, including those in reviews. Because of these efforts, the apps used in this study were also quite homogenous in terms of their ratings (all 15 apps were rated above 4.1 out of 5). Including a broader range of apps (both in terms of greater variation in ratings and in terms of number of users and downloads) may reveal additional challenges users face when using diet-tracking apps.

### Conclusions

Assessment of 72,084 user reviews for diet-tracking apps revealed an overall positive user evaluation. Users highly value the ability to track their food intake and manage their weight. Nonetheless, there is significant room for improvement, particularly in the area of charges associated with app use and features that enable adding food to the apps’ databases. The findings of this study provide relevant insights into user opinions and evaluations of diet-tracking apps.

The implications of this study go beyond those for app developers as stakeholders; for example, in cases concerning health and nutrition, public policy and official institutions should be involved. Digital participation of current and future generations is increasing; there is also evidence that mobile apps are a potentially useful tool for shaping and tracking users’ diets [8,14]. By exploring users’ experiences with apps, along with their suggestions and comments, it is possible to better support the apps they need and improve their eating habits, health and diet management, and nutrition-related well-being.

## Abbreviations

NLP: natural language processing
